# Special Issue “Functional Characterization of Lactic Acid Bacteria”: Editorial

**DOI:** 10.3390/microorganisms11051190

**Published:** 2023-05-01

**Authors:** Franca Rossi

**Affiliations:** Istituto Zooprofilattico Sperimentale dell’Abruzzo e del Molise, Sezione di Campobasso, 86100 Campobasso, Italy; f.rossi@izs.it

Lactic acid bacteria (LAB) are a diverse group of microorganisms of the order *Lactobacillales* in the *Bacillota* phylum, subdivision *Bacilli*, comprising, at this stage of taxonomic descriptions six families (*Aerococcaceae*, *Carnobacteriaceae*, *Enterococcaceae*, *Lactobacillaceae*, *Leuconostocaceae* and *Streptococcaceae*). These bacteria have in common a fermentative metabolism with lactic acid as the main or one of the main final products from carbohydrate utilization. An updated list of genera and species of LAB is available at https://lpsn.dsmz.de/order/lactobacillales (accessed on 3 March 2023). 

The vast majority of LAB species are non-pathogenic, though able to cause opportunistic infections in rare cases [1,2]. These bacteria are responsible for the food and feed natural fermentation processes that allow the transformation of perishable food commodities into more durable products. Safe use since ancient times has led to the recognition of the qualified presumption of safety (QPS) status for many LAB species [3]. Foods and feeds fermented by LAB are safer because of the inhibition exerted by LAB towards pathogenic and deteriorating microorganisms, are sensorially appreciated, are better digestible, have an improved bioavailability of nutrients, and are enriched with bioactive molecules and vitamins synthetized by LAB. In addition, LAB are able to colonize human and animal body districts, and, most importantly, GIT, where they exert health-promoting effects for the host.

However, the multiple desired activities of LAB are expressed at different extents by individual strains, even at the intra-species level, because they depend on the possession of variable genetic characters encoded by unstable genome regions. Horizontal gene transfer events were also responsible sometimes for the acquisition of risk characters, such as genes for antibiotic resistance (AR) and for biogenic amine formation (BA), also by strains belonging to non-pathogenic LAB species [4], and these events must be analyzed at the strain level. It must be also considered that functional features are still little characterized for a large number of the described LAB species, which account for more than two hundred to date.

Therefore, the Special Issue entitled “Functional characterization of lactic acid bacteria” was intended to collect scientific articles regarding the functional characterization of LAB strains in relation to the possession of specific genetic traits and for an advancement of knowledge on the potential of these bacteria in improving human or animal health. Valuable research and review papers were submitted to the Special Issue, which reflects the wide range of functional traits to be considered for this diverse group of bacteria.

Monteiro and Duarte [5] collected the major findings from transcriptome and proteome studies in *Lactococcus lactis* with the overexpression of native or recombinant proteins. Indeed, *L. lactis* is a LAB species traditionally used for the production of proteins or metabolites for application in the food and pharmaceutical industry, for the production of pharmaceutical-grade plasmid DNA for DNA vaccination, and as a live vector for the delivery of DNA, proteins, or metabolites to mucosal surfaces. Information from transcriptome and proteome studies allowed to optimize the yield of products also by giving indications on which genes to remove or overexpress in *L. lactis* strains.

Metabolic engineering strategies were developed for pathways highly represented in the *L. lactis* proteome such as the ones involved in the pyruvate metabolism, to increase the productivity of alanine, a food sweetener, diacetyl, a flavoring compound and molecules for pharmaceutical applications such as vitamins B11 and B2 and hyaluronic acid. Genome reduction in *L. lactis* NZ9000 strain by deletion of nonessential DNA regions using the Cre-loxP deletion system led to obtaining a faster-growing strain and fewer maintenance demands for more efficient recombinant-protein production.

Notably, the overexpression of the cell envelope stress response (CesSR) genes improved the endogenous and heterologous membrane protein production for the use of *L. lactis* as a live mucosal vaccine with efficient recognition by the host immune system.

The study of Yang et al. [6] regarded the identification and functional definition of LAB communities naturally selected in a fermented total mixed ration (FTMR) enriched with oat silage made from oat (*Avena sativa*), a highly nutritious forage produced abundantly in China provinces during the rainy season. Its ensiling allows to preserve this feed for longer but the microbiological dynamics of an FTMR containing oat silage were still unknown. Yang et al. [6] observed that the LAB population increased with the addition of oat silage and microbial diversity analysis based on 16S rRNA sequencing indicated that, while samples not added with oat silage were dominated by the homofermentative species *Lactobacillus acetotolerans*, in those with oat silage heterofermentative species *Levilactobacillus buchneri*, *L. brevis* and *Companilactobacillus versmoldensis* increased and a faster yeast population decline occurred. These events are known to enhance the aerobic stability of silage. In the study, the role of *Loigolactobacillus coryniformis* in the fermentation process was also highlighted.

Widodo et al. [7] elucidated the metabolic pathways involved in the production of butyric acid, a compound with anticarcinogenic and antioxidative properties, by *Lacticaseibacillus casei* AP, isolated from the digestive tract of healthy Indonesian infants, and by *Lactiplantibacillus plantarum* DR131, isolated from indigenous fermented buffalo milk, by genome comparison. It was observed that a butyrate kinase (*buk*) and a phosphotransbutyrylase (*ptb*) in *L. casei* AP and the γ-Aminobutyric acid (GABA) pathway in association with a medium-chain thio-esterase and the type 2 fatty acid synthase (FASII) in *L. plantarum* DR131 are responsible for butyric acid production. Therefore, the presence of these pathways is distinctive of *L. casei* and *L. plantarum* strains able to produce butyric acid.

LAB are non-respiring organisms but carry genes encoding components of the respiratory chain, which is complete in the case of *L. lactis* and *Leuconostoc mesenteroides*, and respiration occurs upon supplementation with heme. This leads to enhanced biomass formation, reduced acidification, resistance to oxygen, and improved long-term storage, relevant for industrial applications. Supplementation with menaquinone (vitamin K2), the sole quinone in the electron transport chain in Gram-positive bacteria, led to a respiratory behavior also in other LAB species, indicating that the respiratory chain can be incomplete for the lack of quinones. Therefore, Watthanasakphuban et al. [8] confirmed the functionality of menaquinone production pathway genes found in the genomes of *L. plantarum* WCFS1 and *L. buchneri* DSM 20057 by complementation in *L. cremoris* knockout mutants for the same genes. *L. plantarum* WCFS1 transformed with a construct comprising the menaquinone biosynthesis genes absent in its genome, controlled by two inducible promoters, produced higher biomass, indicating menaquinone biosynthesis and completion of the respiratory chain in this strain.

Short-chain fructooligosaccharides (FOSs) are defined as prebiotics because they are able to stimulate beneficial gut microbes. However, their effect on the physiology of *Lactobacillus gasseri* and *L. paragasseri*, human commensals comprising well-characterized probiotics, were still little defined. Shiraishi et al. [9] investigated the effect of glucose, kestose, and raffinose on lipoteichoic acid (LTA) production by *L. gasseri* JCM 1131^T^ and *L. paragasseri* JCM 11657. LTA determines several beneficial activities of probiotics, including immune stimulation. *L. gasseri* JCM 1131^T^ and *L. paragasseri* JCM 11657 were found to metabolize kestose but not raffinose, possibly for the presence of one or two specific glycoside hydrolases. LTA production in *L. gasseri*, was at least 15-fold higher in cells cultured with raffinose compared with those cultured with glucose or kestose. Further studies should elucidate the impact of the LTA types produced on the probiotic properties of *L. gasseri* and *L. paragasseri*.

Ruppitsch et al. [10] characterized six *Leuconostoc mesenteroides* isolates from traditional Montenegrin brine cheeses from nine producers by whole genome sequencing (WGS). This species is among the *Leuconostoc* spp. applied as adjunct cultures in food production. In the study, a core genome multilocus sequence typing (cgMLST) scheme comprising 960 core genome target genes and 935 accessory genome target genes was defined on the basis of a genome-wide gene-by-gene comparison also with other 19 genome sequences available in GenBank. The cheese isolates showed a high diversity, possibly explained by origin from diverse plants in contact with animals fed by grazing. This finding underlines the importance of traditional food products as a source for strains with unique features to be used in dairy productions to preserve the designation of origin and to create added-value sensory characteristics.

Pan et al. [11] identified the genes that confer bile salt tolerance, decisive for the survival of probiotics in the gut, to *Ligilactobacillus salivarius* by comparative genomic analysis, verification by quantitative reverse transcriptase PCR (qRT-PCR) analysis in the presence of bile salts and knockout of genes with highest upregulation. The authors screened 90 *L. salivarius* strains isolated from 500 stool samples of Chinese individuals from 17 provinces and only eight strains were able to survive above 9.5% in the presence of 0.3% bile salts, the average concentration in the human intestine. Among these, *L. salivarius* FWXBH36M1 showed the maximum survival rate. All genomes were sequenced and their comparison showed that 15 tolerant core genes, comprising four genes for peptidoglycan synthesis, seven genes for phosphotransferase system (PTS), mostly mannose- and N-acetylglucosamine-specific, and one chaperone-encoding gene, were missing in at least two non-tolerant strains. Moreover, four genes related to DNA damage repair and transport were redundant in the strains with high bile salt tolerance. The intra-strain diversity of bile salt tolerance in *L. salivarius* was not related to the evolutionary variations and presence/absence of the three identified bile salt hydrolases (BSHs). Knockout experiments with *L. salivarius* FWXBH36M1 indicated that the survival rates of mutant strains for the genes mostly upregulated in the presence of bile salts, LSL_1568, LSL_1716, and LSL_1709, were significantly lower than that of the wild-type strain under bile salt conditions. Therefore, the study provided an exhaustive insight into the variability of bile salt tolerance in the *L. salivarius* species and the genetic traits involved, also indicating potential gene targets for the rapid screening of bile salt-tolerant *L. salivarius* strains by PCR amplification.

Li et al. [12] carried out the first genetic analysis on citrate metabolism pathways that lead to the formation of acetate, diacetyl, acetoin, and 2,3-butanediol flavor compounds, for the *Carnobacterium* genus by examining six strains of the species *C. maltaromaticum*, common in milk and soft cheeses, in meat and fish products and known to affect the sensory profile of dairy products. Four strains showed weak citrate utilization, while two strains did not metabolize citrate. Genome sequences showed the presence of two different pathways for citrate utilization: pathway 1, with oxaloacetate as an intermediate and pathway 2 with isocitrate and α-ketoglutarate as intermediates. Pathway 1 was present only in some strains. Further studies should elucidate the conditions that activate the citrate pathways and if citrate catabolism in pathway 1 or 2 may result in the production of flavor compounds in cheeses.

Song et al. [13] investigated the probiotic properties of LAB isolated from Jeot-Gal, a traditional Korean high-salt fermented seafood. In particular, they focused on the production of conjugated linoleic acid (CLA) and immune modulation in *Cenorabditis elegans* as in vivo model for immune-regulatory pathways examination and alternative to human intestinal cell lines. CLA is a mixture of the isomers of linoleic acid cis-9, trans-11-CLA, with immunity enhancing, anticarcinogenic, antiatherogenic, antidiabetic, and anti-obesity activities and trans-10, cis-12-CLA, with energy metabolism-enhancing, lipid reduction, bone health promotion, and growth improvement activities in animal models and diseased human cell lines produced by various LAB, particularly lactobacilli, through enzymatic isomerization. Song et al. [13] selected strains *of L. paraplantarum, L. plantarum, L. pentosus*, *Pediococcus acidilactici* and *L. mesenteroides* that produced between 86.0 and 125.5 μg/mL CLA in different isomer ratios from 0.2% LA in the culture medium. Among these *L. plantarum* JBCC105655, *L. plantarum* JBCC105683, and *P. acidilactici* JBCC105117, stimulated both pro-inflammatory and anti-inflammatory cytokines in *Cenorabditis elegans*, showing to be potentially able to modulate the innate and adaptive immune system and inflammatory response. The use of these LAB of food origin as probiotics is therefore promising and should be further pursued to increase the content of this beneficial compound in fermented products

Bacteria of the *Lacticaseibacillus* genus, mainly *Lacticaseibacillus casei*, *L. paracasei*, *L. rhamnosus*, and *L. zeae* comprise well-characterized probiotics. However, individual strains have a variable capacity to exert beneficial effects, so that it could be useful to fix selection criteria for those with enhanced probiotic potential. An analysis of the fully annotated genomes of these species available from the GenBank database, 99 at the time of analysis, was carried out to identify gene loci encoding for characteristics involved in survival in GIT, adhesion to mucus or other host molecules, and production of cell-surface-associated macromolecules, including exopolysaccharides (EPS), involved in adhesion and immune stimulation [14]. Conserved genes include those encoding for a bile salt hydrolase, three surface antigens, proteins involved in the synthesis of LTA and teichoic acids, in biofilm formation and binding to mucus. Variable traits are genes involved in SpaCBA and SpaFED pili production, adhesion molecules, EPS synthesis, taurine, and fucose utilization. The latter traits were found to be variable by PCR tests in isolates of intestinal origin. Results indicated the feasibility of preliminarily analyze the presence of some variable traits in strains to be evaluated as probiotics but experimental validation should be carried out to confirm the functional role of the identified genetic traits.

This Special Issue offered insights into a varied range of application fields for LAB, from requisites and ability to exert in vivo probiotic activities, to the production of beneficial molecules, feed fermentation improvement, and production of heterologous proteins and metabolites. Research on a wider number of species and strains will make available a wealth of knowledge on how to apply these beneficial bacteria for food, biotechnological, pharmaceutical, and probiotic applications.
